# Climate-Driven Habitat Shifts of Two Palm Squirrel Species (Sciuridae: *Funambulus*) and Projected Expansion of Their Range Overlap with Indian Agroecosystems

**DOI:** 10.3390/biology14121666

**Published:** 2025-11-24

**Authors:** Imon Abedin, Paromit Chatterjee, Hilloljyoti Singha, Hyun-Woo Kim, Shantanu Kundu

**Affiliations:** 1 Wildlife Ecology Lab, Department of Zoology, Bodoland University, Kokrajhar 783370, India; 2 Habitat Lens Private Limited, Khardah 700118, India; 3 Centre for Wildlife Research and Biodiversity Conservation, Bodoland University, Kokrajhar 783370, India; 4 Department of Marine Biology, College of Fisheries Science, Pukyong National University, Busan 48513, Republic of Korea; 5 Marine Integrated Biomedical Technology Center, National Key Research Institutes in Universities, Pukyong National University, Busan 48513, Republic of Korea; 6 Research Center for Marine Integrated Bionics Technology, Pukyong National University, Busan 48513, Republic of Korea; 7 Ocean and Fisheries Development International Cooperation Institute, College of Fisheries Science, Pukyong National University, Busan 48513, Republic of Korea; 8 International Graduate Program of Fisheries Science, Pukyong National University, Busan 48513, Republic of Korea

**Keywords:** rodent, agriculture, pest species, habitat expansion, management strategy, human–wildlife conflict, species distribution model

## Abstract

Ecosystems worldwide are being influenced by climate change in complex ways, leading to profound impacts on biodiversity. While these shifts may constrain the adaptive capacity of some species, they may simultaneously promote the expansion of others, including certain rodents. The *Funambulus pennantii* (five-striped northern palm squirrel) and *Funambulus palmarum* (three-striped palm squirrel) demonstrate exceptional adaptability across diverse habitats from rural plantations to densely populated urban areas. Their close association with humans ranges from causing crop damage to becoming urban nuisances, leading to their broad classification as agricultural pests. Hence, this study utilizes ensemble species distribution models to identify the climatic niches of these two species and delineate overlapping suitable habitats within agricultural zones in current and different future climate scenarios. The ensemble model identified 215,748 km^2^ of suitable habitat for *F. pennantii* and 39,578 km^2^ for *F. palmarum* under current climatic conditions in India. The future projections indicate habitat increases of about 20.6% for *F. pennantii* and 48.1% for *F. palmarum*. The agricultural overlap is also projected to rise by up to 45% and 48%, respectively, compared with present overlaps of 215,595 km^2^ and 39,073 km^2^. The most substantial expansions are projected in the Gangetic Plains, Central Highlands, and parts of the Western and Eastern Ghats and Deccan Plateau. These findings suggest that both species possess high resilience to environmental change and may pose increasing risks to agriculture in a warming climate. The study underscores the need for proactive, ecologically sustainable management strategies to mitigate potential human–squirrel interactions and agricultural losses, offering a foundation for targeted regional interventions.

## Simple Summary

Climate change is reshaping ecosystems and altering the capacity of species to adapt in different landscapes. This study examines two highly adaptable native Indian squirrels (*Funambulus pennantii* and *Funambulus palmarum*), both of which thrive across rural and urban landscapes and are recognized agricultural pests. Using ensemble species distribution models, the study investigates their climatic niches to predict potential habitat shifts under future climate scenarios. The projections reveal that both species are likely to expand their ranges, particularly across major agricultural zones. These results underscore their ecological resilience and emphasize the need for proactive management to address rising human–squirrel conflicts and crop losses.

## 1. Introduction

Climate change is influencing ecosystems worldwide in complex and interconnected ways, with significant consequences for terrestrial biodiversity and profound impacts anticipated in the future [1,2]. The manifestations of climate change include rising temperatures, altered precipitation regimes, and elevated climate variability. Such changes can restructure species dispersals, population dynamics, and ecosystem processes by modifying ecological niche factors such as habitat availability, resource supply, and biotic interactions [2,3,4,5]. While these changes may constrain the ability of some species to adapt to local climatic conditions, leading to range contractions or even extinction, they may also facilitate the expansion of other species, including certain rodents [6,7,8]. Although most rodent species remain confined to natural habitats within their distribution ranges, some have successfully colonized highly disturbed environments, including urban areas. Consequently, many rodent species have emerged as agricultural and urban pests and serve as key reservoirs and vectors of numerous pathogens transmissible to humans and domestic animals [9,10,11,12]. In Asia, rodents are responsible for extensive agricultural losses, posing a major risk to the food security of smallholder farmers [13,14,15]. These impacts are likely driven by the remarkable ecological plasticity and adaptive capacity of certain rodent species in response to climatic and environmental change [16].

The genus *Funambulus*, belonging to the family Sciuridae, comprises a group of small-bodied, diurnal rodents commonly known as palm squirrels [17]. These squirrels are widely distributed across diverse ecological zones, including dry deciduous forests, agricultural fields, urban parks, and forest plantations. Among them, species such as *Funambulus pennantii* (five-striped northern palm squirrel) and *Funambulus palmarum* (three-striped palm squirrel) exhibit remarkable adaptability to habitats ranging from rural plantations to densely populated urban centers [18,19]. Specifically, the *F. pennantii* is distributed broadly across northern and central India, Pakistan, Nepal, and Bangladesh, with its southernmost native range extending approximately to the Godavari River and overlapping into parts of Maharashtra and Karnataka [18,19,20]. In contrast, *F. palmarum* is the dominant palm squirrel of peninsular India and much of Sri Lanka, with its northern distribution generally limited to southern Madhya Pradesh, Odisha, and parts of Gujarat [18,19,21]. Originally forest dwellers, both *F. pennantii* and *F. palmarum* have successfully expanded into farmlands, plantations, forest edges, sacred groves, urban parks, and residential landscapes. Their persistence across such heterogeneous environments demonstrates high behavioral plasticity and resilience to habitat modification [18,19,22]. The relationship between *Funambulus* squirrels and human communities has become increasingly complex due to their expansion into anthropogenic landscapes. As conspicuous and adaptable species thriving in human-modified landscapes, *F. pennantii* and *F. palmarum* are simultaneously subjects of cultural admiration and sources of conflict [23,24,25,26]. The human–squirrel interactions range from crop damage and urban nuisance to their widespread characterization of *Funambulus* squirrels as agricultural pests [27,28,29].

Owing to climatic shifts, rodent range expansions may occur, with far-reaching consequences that negatively affect ecosystems, public health, and food security [30,31,32,33]. Consequently, understanding the projected effects of climate change on rodent pest distributions is essential for effective risk assessment and the development of management interventions [33,34]. The proactive measures to mitigate the adverse impacts of rodent pests are therefore critical to safeguarding both environmental and human health. The urgency of such strategies has become gradually pronounced with the amplifying impacts of global climate change and given their high ecological adaptability and potential to trigger biological invasions to disrupt the stability of recipient ecosystems [35,36]. In this regard, species distribution models (SDMs) are now emerging as empirical tools used to predict the environmental envelope of species in relation to environmental variables [37]. These models apply statistical or machine-learning algorithms to species occurrence data in combination with relevant environmental predictors to simulate suitable habitat distributions across space and time [38]. Their robust performance and reliable assessments have been demonstrated globally and are now widely employed to predict the potential distributions of rodent pests and to conduct associated risk assessments [37,38,39,40,41,42,43]. Hence, the present study aims to: (i) identify the current climatic niche of the two *Funambulus* species; (ii) project their potential distributions under future climate scenarios; and (iii) delineate overlapping suitable habitats within agricultural zones under both present and future conditions. The findings are intended to inform the development of effective management strategies, aligned with national and regional action plans, to mitigate potential conflicts and risks associated with these species.

## 2. Materials and Methods

### 2.1. Study Area and Occurrence Records

**Figure 1:**
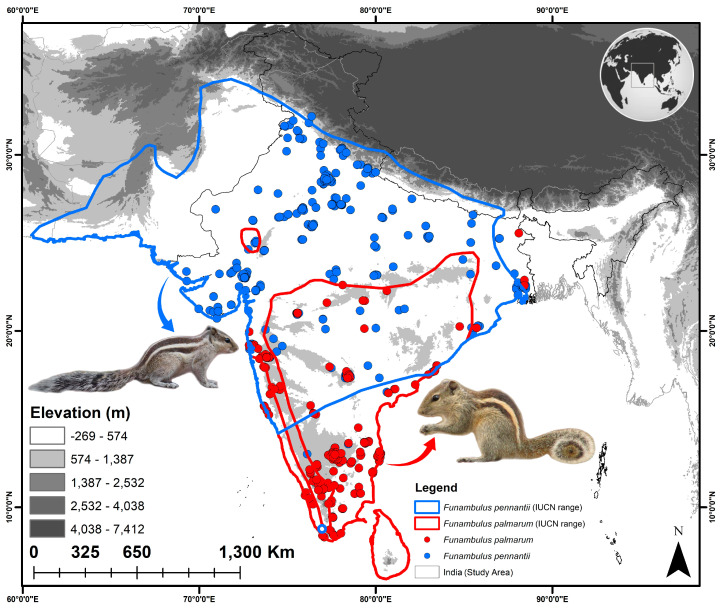
Occurrence records of *F. pennantii* and *F. palmarum* in India, compiled from secondary sources along with their IUCN range. The inset photographs of both species are sourced from the free repository Wikimedia Commons.

Both species are primarily distributed across South Asia, with the majority of their range falling within India. Given this extensive distribution, India was selected as the training range for the study (Figure 1). Moreover, as one of the world’s most agriculturally dependent economies, India provides an important context for assessing potential shifts in rodent distributions under changing climatic scenarios, while also aligning with national policies and regional management plans. The occurrence data for both species in the last five years were obtained from secondary sources using the IUCN Geospatial Conservation Assessment Tool (GeoCAT, https://geocat.iucnredlist.org/, accessed on 5 October 2025) [44]. Further, to diminish spatial autocorrelation and overrepresentation of clustered records, all occurrence points were spatially rarefied at a resolution of 4.5 km^2^ using the rarefaction function in SDM Toolbox v2.4 [45]. The chosen rarefaction scale was consistent with the resolution of the environmental raster layers used in the following analyses. After filtering and rarefaction, 258 and 283 unique occurrence records of *F. pennantii* and *F. palmarum*, respectively, were engaged for habitat modeling.

### 2.2. Modeling Predictors

The study took a combination of climatic and topographic variables to identify the suitable habitat envelope of the species [46]. The available 19 bioclimatic variables were obtained from the WorldClim database available at a spatial resolution of 2.5 min (~4.5 km^2^) that is widely used in SDM studies (https://www.worldclim.org/, accessed on 5 October 2025) [47]. The topographic predictors encompassed elevation, slope, and aspect, resulting from Shuttle Radar Topography Mission (SRTM) data retrieved at 90 m resolution (http://srtm.csi.cgiar.org/srtmdata/, accessed on 5 October 2025). The spatial predictor layers were then resampled and standardized to a resolution of 2.5 min (~4.5 km^2^) using the Spatial Analyst extension in ArcGIS 10.6 to confirm consistency in scale across model inputs. Further, the forthcoming climate projections were analyzed for two Shared Socioeconomic Pathways (SSP245 and SSP585) in two time horizons: 2041–2060 and 2061–2080. The climate data were obtained from the HadGEM3-GC31-LL model of the CMIP6 framework, as this General Circulation Model (GCM) is recognized as one of the best-performing models for South and Southeast Asia [48,49]. Additionally, to identify overlaps between suitable areas and agricultural land-cover data. This land-cover data was obtained from the ESRI Sentinel-2 10 m Land Use/Land Cover (LULC) dataset available on the Living Atlas platform (https://livingatlas.arcgis.com/landcover/, accessed on 5 October 2025) [50]. The variables were tested for pairwise correlations, and those with coefficients exceeding |r| > 0.8 were excluded from further analysis (Figures S1 and S2) [51]. Specifically, three correlation metrics, viz. Pearson, Spearman, and Kendall, were computed using the SAHM (Software for Assisted Habitat Modeling) package in the VisTrails version 2.2.3 platform [52]. If the correlation between any two variables exceeded the threshold in any of the tests, one of the variables was excluded from the final selection based on its importance as assessed by the package. Following this screening process, nine and eight uncorrelated variables were retained for the final habitat suitability modeling of *F. pennantii* and *F. palmarum*, respectively.

### 2.3. Ensemble Distribution Model

An ensemble framework, which integrates multiple algorithms, was employed to build inclusive and robust distribution models for both target species. This approach utilizes the complementary strengths of each algorithm and captures the broader aspects of ecological relationships [53]. In this study, five algorithms used within the ensemble framework were Boosted Regression Tree (BRT), Generalized Linear Model (GLM), Multivariate Adaptive Regression Splines (MARS), Maximum Entropy (MaxEnt), and Random Forest (RF) [38,54,55]. The ensemble modeling was conducted using the Software for Assisted Habitat Modeling (SAHM) integrated in the VisTrails workflow system [52,56]. The resulting outputs generated continuous predicted habitat suitability maps that ranged from 0 (unsuitable) to 1 (highly suitable). The outputs were converted into binary presence–absence via the sensitivity-equals-specificity (SES) threshold. Specific models with an area under the receiver operating characteristic curve (AUC) greater than 0.75 were taken [57]. The ensemble model agreement map was then produced, with pixel values ranging from 0 to 5, reflecting the number of participating algorithms in predicting each location as suitable habitat. The value of 5 indicated complete consensus between each algorithm. Additionally, the model performance was evaluated using multiple metrics such as AUC, True Skill Statistic (TSS), Cohen’s Kappa, Proportion Correctly Classified (PCC), sensitivity, and specificity. These metrics were considered for both training datasets and across 10-fold cross-validation replicates to ensure model robustness and reliability [58,59,60,61].

## 3. Results

### 3.1. Model Evaluation

**Figure 2:**
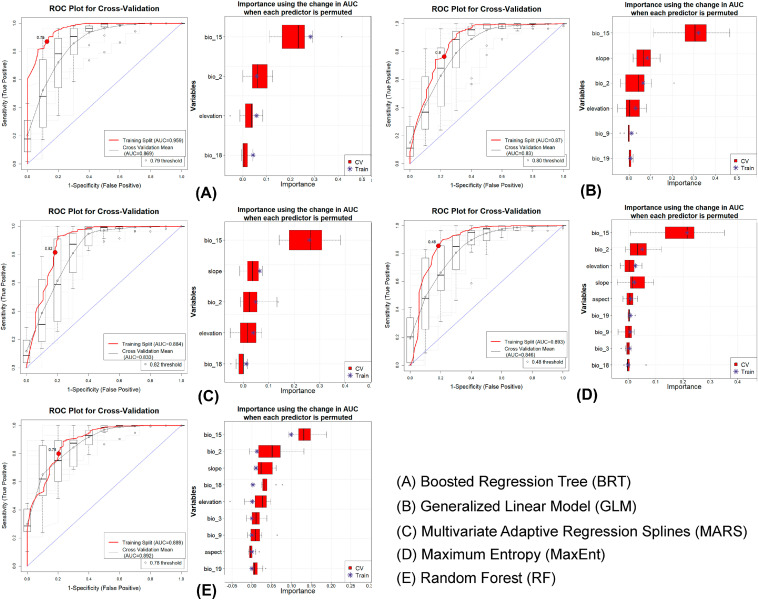
Model performance and variable importance for *F. pennantii* generated using the five algorithms included in the ensemble SDM: (**A**) BRT, (**B**) GLM, (**C**) MARS, (**D**) MaxEnt, and (**E**) RF. In each panel, the left plot shows the ROC curves and corresponding AUC values for both training and cross-validation datasets, while the right plot displays variable importance, indicating the relative contribution of each environmental predictor to the model.

**Figure 3:**
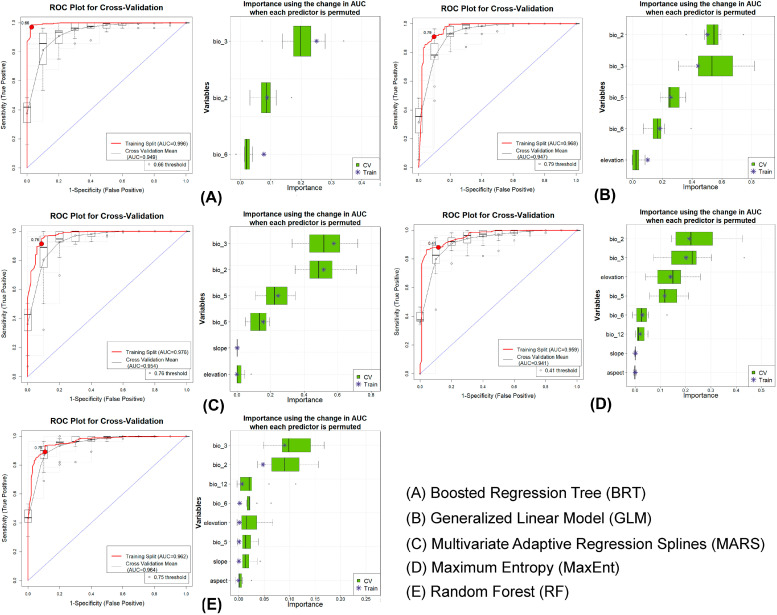
Model performance and variable importance for *F. palmarum* generated using the five algorithms included in the ensemble SDM: (**A**) BRT, (**B**) GLM, (**C**) MARS, (**D**) MaxEnt, and (**E**) RF. In each panel, the left plot shows the ROC curves and corresponding AUC values for both training and cross-validation datasets, while the right plot displays variable importance, indicating the relative contribution of each environmental predictor to the model.

The ensemble model confirmed strong performance for both species in the training and cross-validation sets (Figure 2 and Figure 3). During the training phase, the BRT algorithm yielded the highest AUC values, with 0.959 for *F. pennantii* and 0.996 for *F. palmarum* (Table 1). In contrast, the lowest AUC values were obtained from the RF model for *F. pennantii* (0.884) and from the MaxEnt model for *F. palmarum* (0.959). In the cross-validation phase, the highest AUC values were produced by the RF model for both species, with 0.892 for *F. pennantii* and 0.964 for *F. palmarum* (Table 1). The lowest AUC values in this phase were observed for the GLM model for *F. pennantii* (0.830) and the MaxEnt model for *F. palmarum* (0.941). The analysis of ΔAUC further revealed that the BRT model exhibited the highest variability between the training and cross-validation sets, with values of 0.090 for *F. pennantii* and 0.047 for *F. palmarum*. Conversely, the lowest ΔAUC values were recorded for the RF model, at 0.003 and 0.002 for *F. pennantii* and *F. palmarum*, respectively. Importantly, other evaluation metrics also indicated consistently high performance of the ensemble models across both training and cross-validation datasets for the two *Funambulus* species (Table 1).

### 3.2. Predictor Importance and Response

The ensemble model highlighted distinct climatic and topographic drivers influencing habitat suitability for the two *Funambulus* species (Table 2). Specifically for *F. pennantii*, bioclimatic variables were the strongest predictors, with precipitation seasonality (bio_15) emerging as the most influential factor that accounted for 63.9% of the overall model contribution. This was followed by mean diurnal temperature range (bio_2; 12.6%), slope (9.8%), and elevation (8.3%). The response curves showed that suitability increased sharply at intermediate values of bio_15 but declined at both low and high extremes (Figure S3, Table 2). Similarly, moderate values of bio_2 and slope corresponded with higher occurrence probabilities, while elevation exhibited a unimodal response, with suitability peaking at mid-altitudes. For *F. palmarum*, temperature-related variables were dominant, as isothermality (bio_3) was the most influential predictor (36.6%), followed closely by mean diurnal temperature range (bio_2; 32.1%) and maximum temperature of the warmest month (bio_5; 14.5%). Further, the response curves revealed that habitat suitability increased under intermediate values of bio_2 and bio_3, with suitability decreasing toward both extremes (Figure S4, Table 2). Likewise, bio_5 and bio_6 displayed unimodal responses, with the highest suitability under moderate temperature conditions. Notably, the elevation showed only a weak positive effect at mid-range altitudes, while slope and precipitation contributed minimally to the predictions.

### 3.3. Habitat Suitability: Present and Future

**Figure 4:**
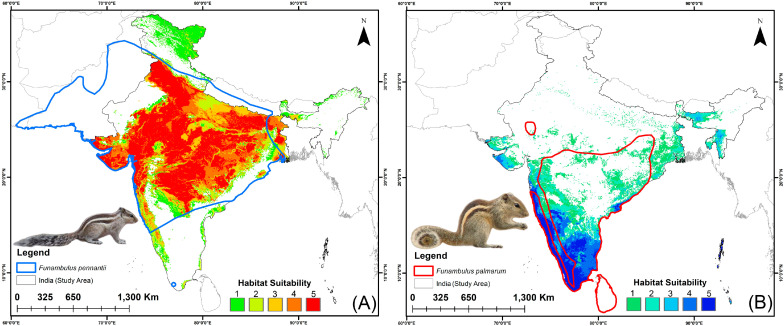
Predicted suitable habitats of *Funambulus* species within India in the present scenario. (**A**) *F. pennantii*; (**B**) *F. palmarum*. Maps illustrate model agreement levels from the ensemble approach, with values ranging up to 5 (full agreement among all five models). Areas with a value of 5 indicate suitable habitats.

**Figure 5:**
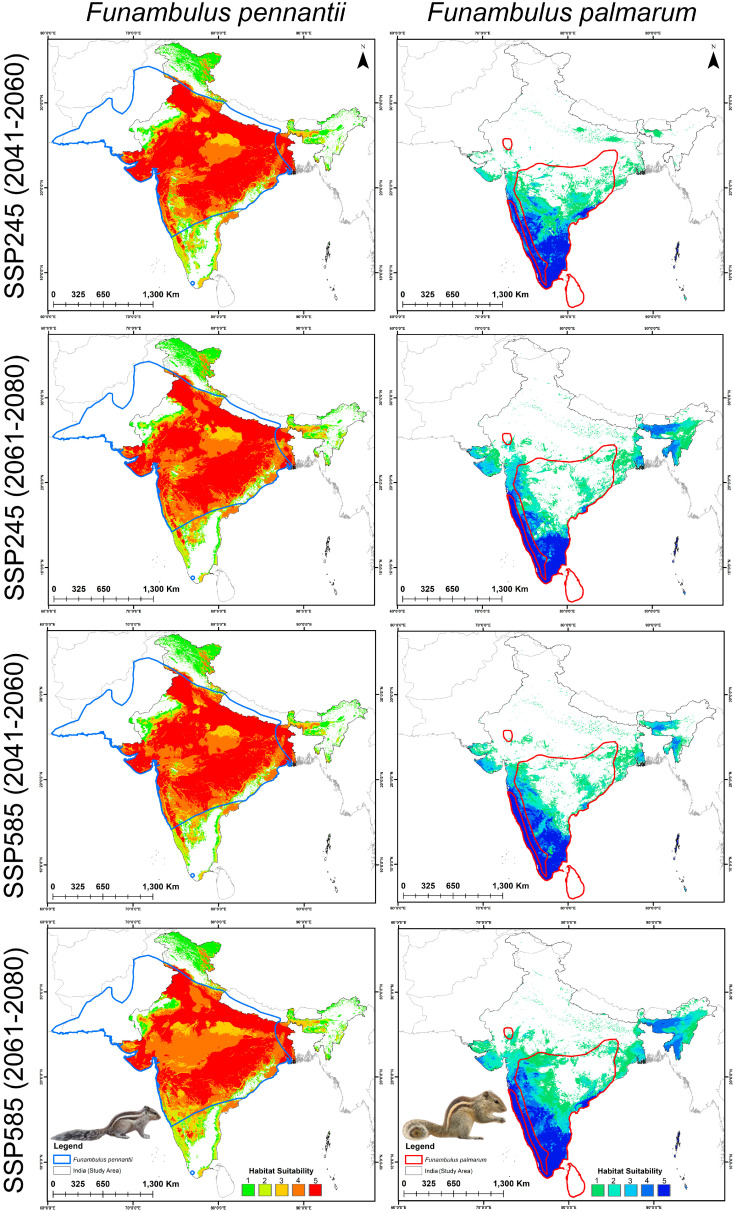
Predicted suitable habitats of *F. pennantii* and *F. palmarum* under future climate scenarios. Maps show projections for two Shared Socioeconomic Pathways, viz. SSP245 and SSP585, for the periods 2041–2060 and 2061–2080.

The ensemble model identified 215,748 km^2^ of suitable habitat for *F. pennantii* and 39,578 km^2^ for *F. palmarum* under the current scenario within India (Figure 4A,B, Table S1). The suitable areas for *F. pennantii* are largely distributed across western, eastern, and central India, encompassing parts of the Gangetic Plains and Central Highlands and extending into the Deccan Plateau. In contrast, the distribution of *F. palmarum* is more restricted and primarily confined to southern India, particularly within the Western and Eastern Ghats, as well as the Konkan and Malabar coasts. The future projections specify an overall increase in suitable habitat for both *Funambulus* species under changed climatic scenarios and time periods due to the ongoing climate change (Figure 5, Table S1). Specifically for *F. pennantii*, habitat suitability is anticipated to increase by 20.557% to 45.513%, whereas for *F. palmarum*, the increase is expected to exceed 48.050%. Under the SSP245 scenario, both species show the greatest expansion during 2041–2060 compared to 2061–2080, with projected increases of 45.513% and 35.487% for *F. pennantii* and 66.230% and 48.050% for *F. palmarum*, respectively. In the SSP585 scenario, a similar trend is observed for *F. pennantii*, with the highest increase of 45.236% occurring in 2041–2060, followed by 20.557% in 2061–2080. However, in the case of *F. palmarum*, the suitability nearly doubles by 2061–2080, while showing a 59.965% increase in 2041–2060 compared to the present. Specifically for *F. pennantii*, a substantial increase in suitable habitat is projected within the semi-arid regions, Konkan Coast, Western Ghats, Central Highlands, and Gangetic Plains. The most pronounced expansion occurs toward eastern India, particularly across the Central Highlands, Gangetic Plains, and parts of the Deccan Plateau. In contrast, *F. palmarum* remains more restricted to southern India, with its projected suitable range concentrated along the Konkan Coast, Western and Eastern Ghats, Coromandel Coast, and parts of the Deccan Plateau. The most substantial increase in habitat suitability for this species is observed along the Eastern Ghats, Coromandel Coast, and southwestern regions of the Deccan Plateau. Nonetheless, both *Funambulus* species are expected to experience an expansion of climatically suitable areas across India in the future relative to the present.

### 3.4. Agricultural Vulnerability: Present and Future

**Figure 6:**
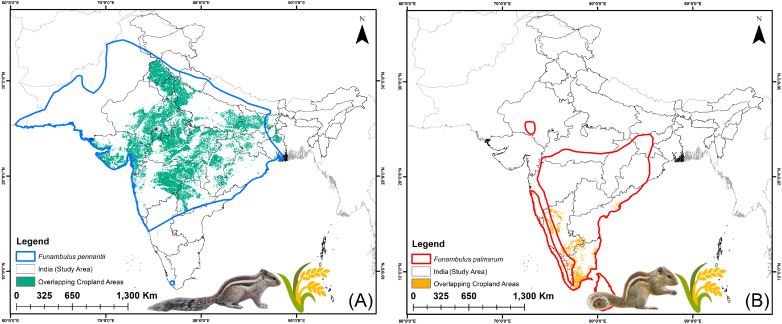
Predicted overlap of suitable habitats with agricultural land for *Funambulus* species within India in the present scenario. (**A**) *F. pennantii*; (**B**) *F. palmarum*.

**Figure 7:**
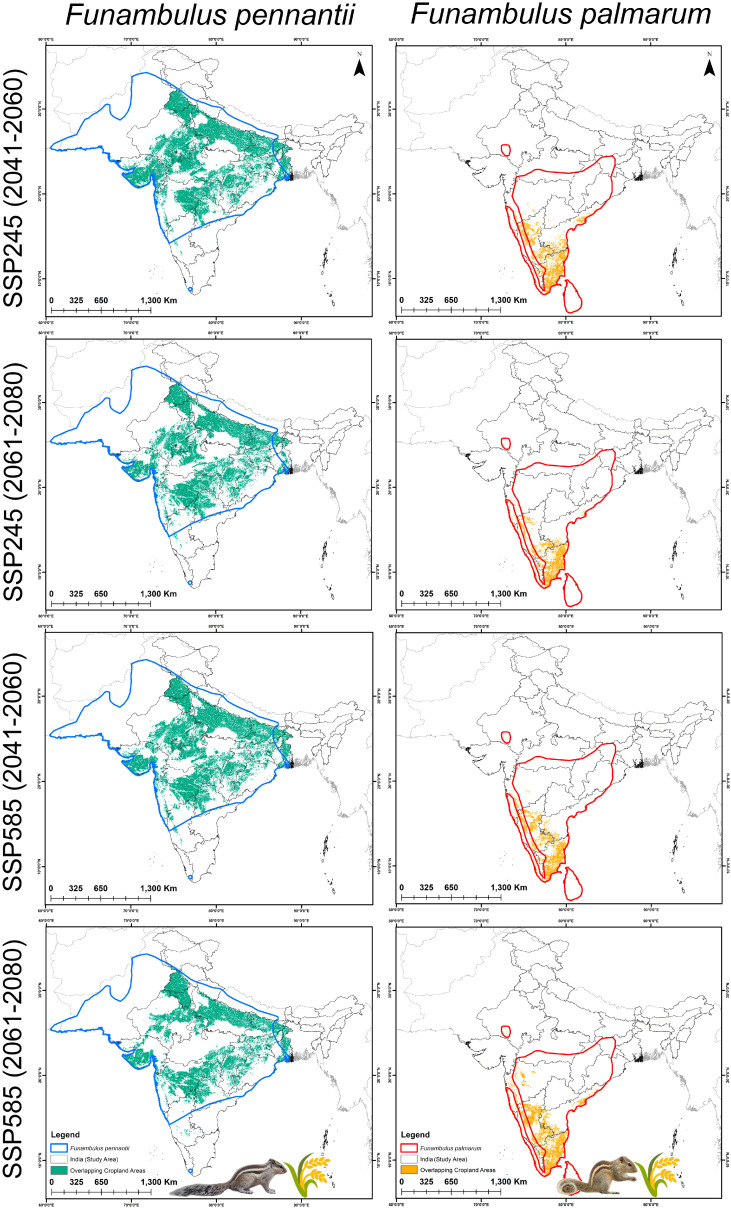
Predicted overlap of suitable habitats with agricultural land for *F. pennantii* and *F. palmarum* under future climate scenarios. Maps show projections for two Shared Socioeconomic Pathways, SSP245 and SSP585, for the periods 2041–2060 and 2061–2080.

**Figure 8:**
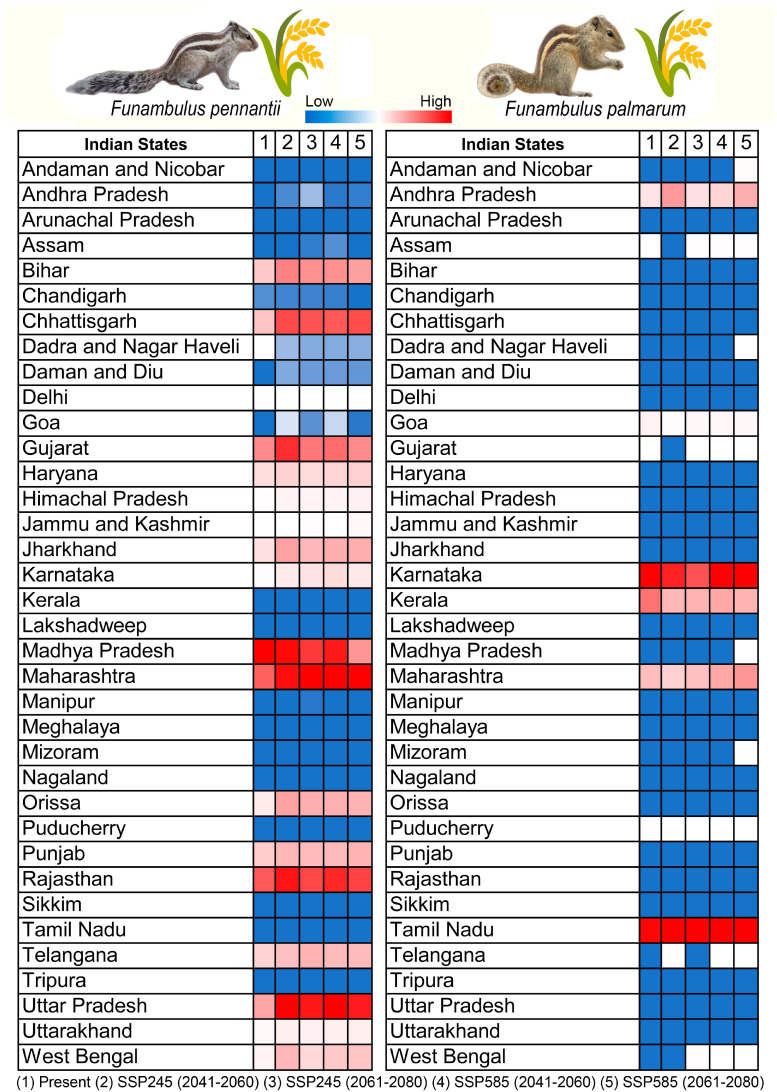
State-level vulnerability assessment of suitable habitats overlapping with agricultural land cover for *F. pennantii* and *F. palmarum* under present and future scenarios. Future projections are shown for two Shared Socioeconomic Pathways, viz. SSP245 and SSP585, for the periods 2041–2060 and 2061–2080.

The suitable habitats overlapping with agricultural areas for both *Funambulus* species represent regions of potential concern. Under the present climatic scenario, *F. pennantii* overlaps with approximately 215,595 km^2^ of agricultural land, while *F. palmarum* overlaps with about 39,073 km^2^ (Figure 6A,B). The most vulnerable states for agricultural areas at risk from *F. pennantii* are Madhya Pradesh (48,762 km^2^), followed by Rajasthan (31,972 km^2^) and Maharashtra (30,573 km^2^) (Table S2). For *F. palmarum*, the states with the highest agricultural overlap are Karnataka (13,261 km^2^), Tamil Nadu (12,591 km^2^), and Kerala (7416 km^2^) (Table S3). Under future climatic scenarios, the suitable habitats overlapping with agricultural lands are projected to increase, reflecting the overall expansion of suitable habitat for both species (Figure 7 and Figure 8). Specifically, for *F. pennantii*, the overlap is expected to increase by up to 45%, while for *F. palmarum*, it is projected to rise by over 48% compared to the present scenario. This increase is most pronounced in the Gangetic Plains, Central Highlands, parts of the Western Ghats, and the Deccan Plateau, with the most vulnerable states for *F. pennantii* projected to be Uttar Pradesh, Maharashtra, and Madhya Pradesh under future scenarios. In contrast, for *F. palmarum*, the expansion of suitable habitats overlapping with agricultural land cover is more evident in the Deccan Plateau and the Eastern and Western Ghats, with states such as Tamil Nadu, Karnataka, Andhra Pradesh, and Kerala showing the highest vulnerability across both future time periods. Hence, these central and southern states of India are likely to remain highly vulnerable to agricultural invasion by the two *Funambulus* species in the future due to climate-driven shifts in habitat suitability.

## 4. Discussion

Global warming is currently evident across the majority of the Earth’s surface, and climate projections indicate a substantial intensification of warming in the forthcoming decades [62,63]. Global climate change is driving the redistribution of life on Earth, with species responding to changing environmental conditions by shifting their geographic ranges [64]. The most evident responses involve movements to higher latitudes, greater elevations, or expansion into newly suitable areas in response to rising temperatures [65,66]. While many species experience reductions in habitat suitability, some taxa, particularly generalist species capable of surviving in a wide range of environments, may show range expansion under future climatic scenarios [67,68,69]. Such expansions are often associated with the colonization of new areas over time, reflecting both ecological flexibility and adaptation to changing conditions. In this context, it becomes essential to conduct studies to assess the range dynamics of species, particularly when range expansions may lead to economic vulnerabilities or increased human–wildlife conflicts [41].

Global redistribution patterns across the family *Sciuridae* indicate that climate change is driving pronounced poleward and elevational shifts, often accompanied by habitat fragmentation and contraction of core ranges [70]. A recent global rapid assessment by Steiner and Huettmann [71] further demonstrated widespread distributional changes among more than 230 squirrel species, including several critically endangered taxa, highlighting that earlier predictive models likely underestimated the extent of these shifts. Their projections identified emerging conservation hotspots across Central and Southeast Asia regions that correspond with the climatic favorability trends observed for the Indian *Funambulus* species in the present study. These findings collectively reinforce that squirrels rank among the most climate-sensitive mammalian groups worldwide. Building on this global context, the present analysis examines the projected range dynamics of *F. pennantii* and *F. palmarum* under different climate change scenarios, documenting their potential habitat expansion and overlap with agricultural land cover. Such insights are vital for anticipating ecological and economic impacts and for developing adaptive management strategies that prioritize vulnerable regions.

The ensemble model identified approximately 215,748 km^2^ of suitable habitat for *F. pennantii* and 39,578 km^2^ for *F. palmarum*, which are projected to increase under future climate scenarios. These expansions and shifts are primarily concentrated across the Gangetic Plains, Central Highlands, Deccan Plateau, and the Western and Eastern Ghats. This could be attributed to the fact that these regions may become warmer with moderately intensified monsoonal conditions in the future, which may favor the persistence of generalist species occurring there [2,72,73,74]. Furthermore, the heterogeneous landscape structure and diverse land-use patterns in these areas may provide ecological opportunities that enable the more adaptable *Funambulus* species to persist and expand, unlike in more environmentally extreme regions [75]. The observed expansions further support the notion that both species are ecological generalists, capable of thriving across a range of environmental conditions and potentially exhibiting climatic resilience [24,28]. This inference is reinforced by the variable importance results, which highlight key climatic predictors influencing habitat suitability. Specifically for *F. pennantii*, precipitation seasonality (bio_15) was the most influential variable, contributing 63.9% to the overall model performance, whereas for *F. palmarum*, isothermality (bio_3) accounted for 36.6%. These findings are consistent with earlier studies on other rodent species, where similar climatic parameters have been shown to facilitate colonization and establishment in newly suitable habitats [41,63]. Since both *Funambulus* species are well adapted to diverse climatic zones, possess broad opportunistic diets, and tolerate a wide range of habitat types, they pose an imminent threat to agricultural ecosystems [76].

With the projected expansion of suitable habitats for both species under future climatic scenarios, a concurrent increase in their overlap with agricultural land cover is also anticipated. Specifically, for *F. pennantii*, this overlap is expected to rise by up to 45%, while for *F. palmarum*, it may increase by over 48% compared to the present scenario. The most vulnerable states to potential invasion include Madhya Pradesh, Rajasthan, Karnataka, Tamil Nadu, and Uttar Pradesh, where a large proportion of the population depends heavily on an agriculture-based economy [77]. Comparable SDM-based studies on invasive squirrels in Europe show that climate change is likely to expand climatically suitable areas and elevate invasion risk for several species, including the eastern gray squirrel and other alien taxa [78,79]. Similar projections for the Barbary ground squirrel indicate a substantial future range expansion under climate change, raising concern about new regions at risk of invasion [80]. Together with broader evidence that climate change will enhance the distribution and pest potential of agricultural rodents globally, these findings support our inference that projected range expansion of *F. pennantii* and *F. palmarum* into croplands could translate into increased economic risk for agriculture in India [2,41]. This poses a major concern, as both species are well-documented agricultural pests known to damage crop fields, plant nurseries, and orchards [81]. They have also been recognized as serious pests of several economically important plantation crops. Due to their high pest potential, *Funambulus* squirrels are considered biosecurity risks and have been categorized as prohibited invasive species in some regions [76]. Given that these squirrels are native to the Indian subcontinent, complete eradication is neither feasible nor ecologically desirable, as they contribute to certain ecosystem services. However, their population expansion and range shifts may intensify human–wildlife conflicts and agricultural losses. Therefore, it is imperative to develop and implement non-lethal, ecologically sustainable management strategies to mitigate their agricultural impacts, as also recommended by previous studies [24,81,82].

The projected range expansion of *F. pennantii* and *F. palmarum* under future climate scenarios represents more than an adaptive shift. It reflects an emergent ecological transformation within India’s agricultural landscapes. These species illustrate how native generalists can become functionally invasive under accelerating climate change, reshaping the structure and dynamics of human-dominated ecosystems. Their expansion is not merely a consequence of warming trends but the outcome of complex feedbacks among climate, land-use intensification, and behavioral plasticity of the species. Such interactions underline a broader transition toward biotic homogenization, where adaptive species progressively dominate at the expense of ecological heterogeneity. The present study therefore highlights an urgent need to integrate predictive ecological modeling with landscape-level biosecurity and sustainable agriculture policies. Anticipating the dual roles of such species, as both ecosystem participants and agricultural competitors, will be essential for maintaining resilience in rapidly transforming agroecosystems and for advancing climate-adaptive management strategies that balance biodiversity conservation with food security.

## 5. Recommendations for Management Interventions

The present study highlights that both *F. pennantii* and *F. palmarum* are projected to expand their suitable habitats across much of India’s semi-arid and humid agricultural zones under future climate scenarios. The predicted increase in range suitability, particularly across central and southern India, aligns with the country’s major grain and fruit-producing landscapes, indicating a potential escalation in crop depredation intensity. Past estimates indicate that small mammals, including squirrels, contribute to annual grain losses ranging between 2.5 and 15 percent, translating to nearly 26 million tonnes or about ₹33,000–35,000 crore in economic terms across Indian farmlands [83]. Although precise estimates for palm squirrels are limited, their adaptive foraging in orchards, nurseries, and household gardens suggests rising economic relevance under shifting climates. The spatial overlap between predicted suitable habitats and intensively farmed areas in Madhya Pradesh, Maharashtra, Rajasthan, and Tamil Nadu highlights an urgent need to integrate these findings into state-level crop management and pest control strategies. India’s National Mission on Sustainable Agriculture (NMSA) and National Adaptation Fund for Climate Change (NAFCC) emphasize climate-resilient production systems, and incorporating species distribution models into these frameworks can enhance early warning mechanisms and adaptive pest management capacity.

Based on these projections, three management priorities emerge, i.e., in high-overlap states such as Madhya Pradesh and Rajasthan, where expansion coincides with open-field cereal systems, early-warning models linked to Krishi Vigyan Kendras should be deployed for pre-sowing advisories. Secondly, in perennial cropping zones of Tamil Nadu and Maharashtra, where *F. palmarum* suitability increases most sharply, adaptive orchard management, through vegetative barriers, diversified canopy structures, and non-lethal deterrence, should be prioritized. Thirdly, in emerging sympatric zones of central India, coordinated monitoring under the National Mission on Sustainable Agriculture (NMSA) can help evaluate competitive and pest dynamics between both species.

Thus, embedding such model-based pest forecasts within the frameworks of the National Adaptation Fund for Climate Change (NAFCC) and state-level programs like the Rajasthan Agri Business Policy and Maharashtra’s Project on Climate-Resilient Agriculture would transform pest management from reactive to predictive. The integration of SDM-derived suitability maps with remote sensing and AI-based forecasting systems offers a pathway to climate-smart pest governance, ensuring that the same data used to project climate risk also directly informs mitigation at the district level.

## 6. Limitations

The present study has several inherent limitations that offer valuable directions for future research. The analysis utilized only bioclimatic and topographic variables, which, although informative, may not fully represent the multifactorial nature of habitat or distribution patterns. Incorporating additional variables such as land-cover dynamics could enhance the robustness of future projections, especially considering that land cover is expected to undergo significant changes in the coming years and may not be accurately captured by static models. Moreover, while this study identified potential climate refugia under present and future conditions and examined their overlap with agricultural land cover, it did not account for the temporal variability or transformation of these agricultural areas. Incorporating the dynamics of agricultural land-use change would therefore provide more timely and realistic insights for management and conservation planning. Additionally, as this research represents a nationwide assessment, regional heterogeneity and local environmental dynamics may not have been fully captured. Therefore, conducting similar studies at regional scales could yield more spatially explicit and policy-relevant outcomes. Furthermore, the study relied on a single GCM for future climate projections, which may limit the comprehensiveness of the results. Therefore, incorporating multiple GCMs and emission scenarios in future assessments would better capture model uncertainty and provide a more robust basis for understanding potential future changes. Building on the foundation established by this study, subsequent research should emphasize region-specific assessments to more effectively account for localized variations and enhance the spatial precision of the results.

## 7. Conclusions

Climate change is altering species distributions globally, with generalist taxa often expanding into newly suitable areas. This trend is evident in the two *Funambulus* species native to India, which are, in some cases, considered agricultural pests. In this study, ensemble modeling identified the current suitable niches of *F. pennantii* and *F. palmarum* within India and projected substantial increases in their suitable range under multiple future climate scenarios. These expansions are likely driven by climatic parameters and the inherent ecological adaptability of these species. Moreover, the anticipated spread of suitable habitats into agricultural regions, particularly in central and southern India, underscores the potential for heightened crop damage and increased human–wildlife conflicts. These findings highlight the need for proactive, ecologically sustainable management strategies that mitigate agricultural impacts while preserving the species’ ecological functions.

## Figures and Tables

**Table 1:** Model evaluation and performance of the algorithms within the ensemble distribution model framework for *F. pennantii* and *F. palmarum* in both training and cross-validation datasets.

Species	Model	Dataset	AUC	ΔAUC	PCC	TSS	Kappa	Specificity	Sensitivity
*F. pennantii*	BRT	Train	0.959	0.090	87.100	0.744	0.693	0.874	0.871
		CV	0.869		81.200	0.592	0.550	0.760	0.832
	GLM	Train	0.870	0.040	76.900	0.542	0.477	0.777	0.766
		CV	0.830		75.300	0.506	0.447	0.751	0.755
	MARS	Train	0.884	0.051	81.500	0.630	0.570	0.816	0.815
		CV	0.833		80.500	0.595	0.549	0.780	0.815
	MaxEnt	Train	0.893	0.047	84.500	0.672	0.629	0.816	0.856
		CV	0.846		79.400	0.568	0.522	0.760	0.808
	RF	Train	0.889	0.003	79.900	0.597	0.537	0.796	0.801
		CV	0.892		86.100	0.596	0.622	0.663	0.934
*F. palmarum*	BRT	Train	0.996	0.047	97.100	0.942	0.928	0.971	0.971
		CV	0.949		87.200	0.718	0.693	0.833	0.885
	GLM	Train	0.968	0.021	90.800	0.813	0.777	0.903	0.910
		CV	0.947		88.200	0.741	0.713	0.845	0.896
	MARS	Train	0.976	0.022	91.300	0.826	0.790	0.913	0.914
		CV	0.954		90.800	0.806	0.774	0.892	0.914
	MaxEnt	Train	0.959	0.018	88.200	0.764	0.718	0.883	0.881
		CV	0.941		87.100	0.732	0.692	0.854	0.878
	RF	Train	0.962	0.002	89.200	0.785	0.742	0.893	0.892
		CV	0.964		90.300	0.712	0.737	0.755	0.957

**Table 2:** Mean (μ) percentage contribution of each environmental variable in the ensemble modeling framework, along with variable importance from each algorithm, for *F. pennantii* and *F. palmarum*. Mean Diurnal Range: bio_2; Isothermality: bio_3; Max Temperature of Warmest Month: bio_5; Min Temperature of Coldest Month: bio_6; Mean Temperature of Driest Quarter: bio_9; Annual Precipitation: bio_12; Precipitation Seasonality: bio_15; Precipitation of Warmest Quarter: bio_18; Precipitation of Coldest Quarter: bio_19; Elevation: elevation; Aspect: aspect; Slope: slope.

Species	Variables	BRT	GLM	MARS	MAXENT	RF	μ (Mean)	μ (Mean) %
*F. pennantii*	Aspect	0.000	0.000	0.000	0.008	0.000	0.002	0.449
	bio_15	0.283	0.321	0.259	0.215	0.099	0.235	63.921
	bio_18	0.043	0.000	0.011	0.001	0.003	0.012	3.166
	bio_19	0.000	0.006	0.000	0.007	0.000	0.003	0.695
	bio_2	0.058	0.064	0.048	0.050	0.013	0.047	12.649
	bio_3	0.000	0.000	0.000	0.002	0.000	0.001	0.137
	bio_9	0.000	0.011	0.000	0.006	0.000	0.003	0.924
	Elevation	0.057	0.030	0.039	0.026	0.001	0.030	8.271
	Slope	0.000	0.084	0.064	0.022	0.011	0.036	9.789
*F. palmarum*	Aspect	0.000	0.000	0.000	0.000	0.000	0.000	0.002
	bio_12	0.000	0.000	0.000	0.020	0.007	0.005	0.625
	bio_2	0.088	0.504	0.517	0.214	0.048	0.274	32.121
	bio_3	0.251	0.440	0.577	0.201	0.090	0.312	36.559
	bio_5	0.000	0.256	0.244	0.116	0.001	0.124	14.479
	bio_6	0.077	0.182	0.156	0.029	0.002	0.089	10.446
	Elevation	0.000	0.102	0.000	0.140	0.001	0.049	5.702
	Slope	0.000	0.000	0.001	0.001	0.000	0.001	0.067

## Data Availability

The data supporting the findings of this study are included within the article and its Supplementary Materials. Additional data or information can be made available upon reasonable request to the corresponding author.

## Supplementary Materials

The following supporting information can be downloaded at https://www.mdpi.com/article/10.3390/biology14121666/s1, Figure S1. Correlation matrix of covariates selected for the final model of *Funambulus pennantii* (r < 0.8). The Pearson correlation coefficient is primarily shown. If the Spearman or Kendall correlation exceeds the Pearson value, an “s” or “k,” respectively, is displayed in the bottom-right corner of the variable box. Figure S2. Correlation matrix of covariates selected for the final model of *Funambulus palmarum* (r < 0.8). The Pearson correlation coefficient is primarily shown. If the Spearman or Kendall correlation exceeds the Pearson value, an “s” or “k,” respectively, is displayed in the bottom-right corner of the variable box. Figure S3. Response curves of each algorithm used in the ensemble model for *Funambulus pennantii*. Each curve illustrates the relationship between predicted habitat suitability and key environmental predictors, as derived from individual algorithms contributing to the ensemble. Figure S4. Response curves of each algorithm used in the ensemble model for *Funambulus palmarum*. Each curve illustrates the relationship between predicted habitat suitability and key environmental predictors, as derived from individual algorithms contributing to the ensemble. Table S1. Suitable habitat area (km^2^) of *Funambulus pennantii* and *Funambulus palmarum* under present and future climatic scenarios. Table S2. Indian state-wise area of suitable habitats overlapping with agricultural land for *Funambulus pennantii* under present and future climatic scenarios. Table S3. Indian state-wise area of suitable habitats overlapping with agricultural land for *Funambulus palmarum* under present and future climatic scenarios.
