# Supplementary material for: Climate-Driven Habitat Shifts of Two Palm Squirrel Species (Sciuridae: Funambulus) and Projected Expansion of Their Range Overlap with Indian Agroecosystems
Source: Biology (Basel). 2025 Nov 24;14(12):1666. doi: 10.3390/biology14121666 (PMC12730107; doi:10.3390/biology14121666)
Supplement: Supplementary file 1 [file biology-14-01666-s001.zip › biology-3952400-supplementary.pdf]

Supplementary Materials

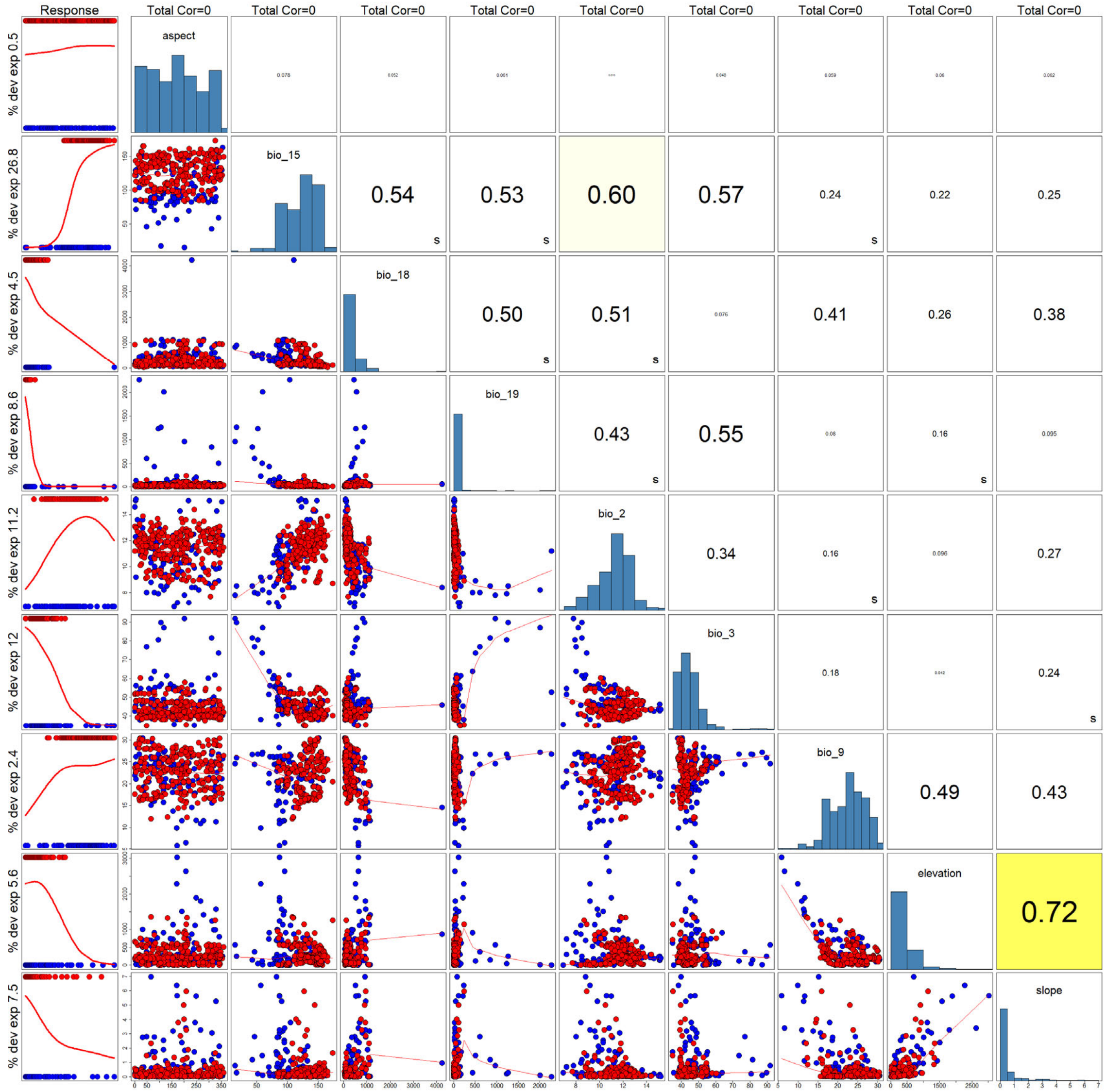

Figure S1. Correlation matrix of covariates selected for the final model of *F. pennantii* (r < 0.8). The Pearson correlation coefficient is primarily shown. If the Spearman or Kendall correlation exceeds the Pearson value, an “s” or “k,” respectively, is displayed in the bottom-right corner of the variable box.

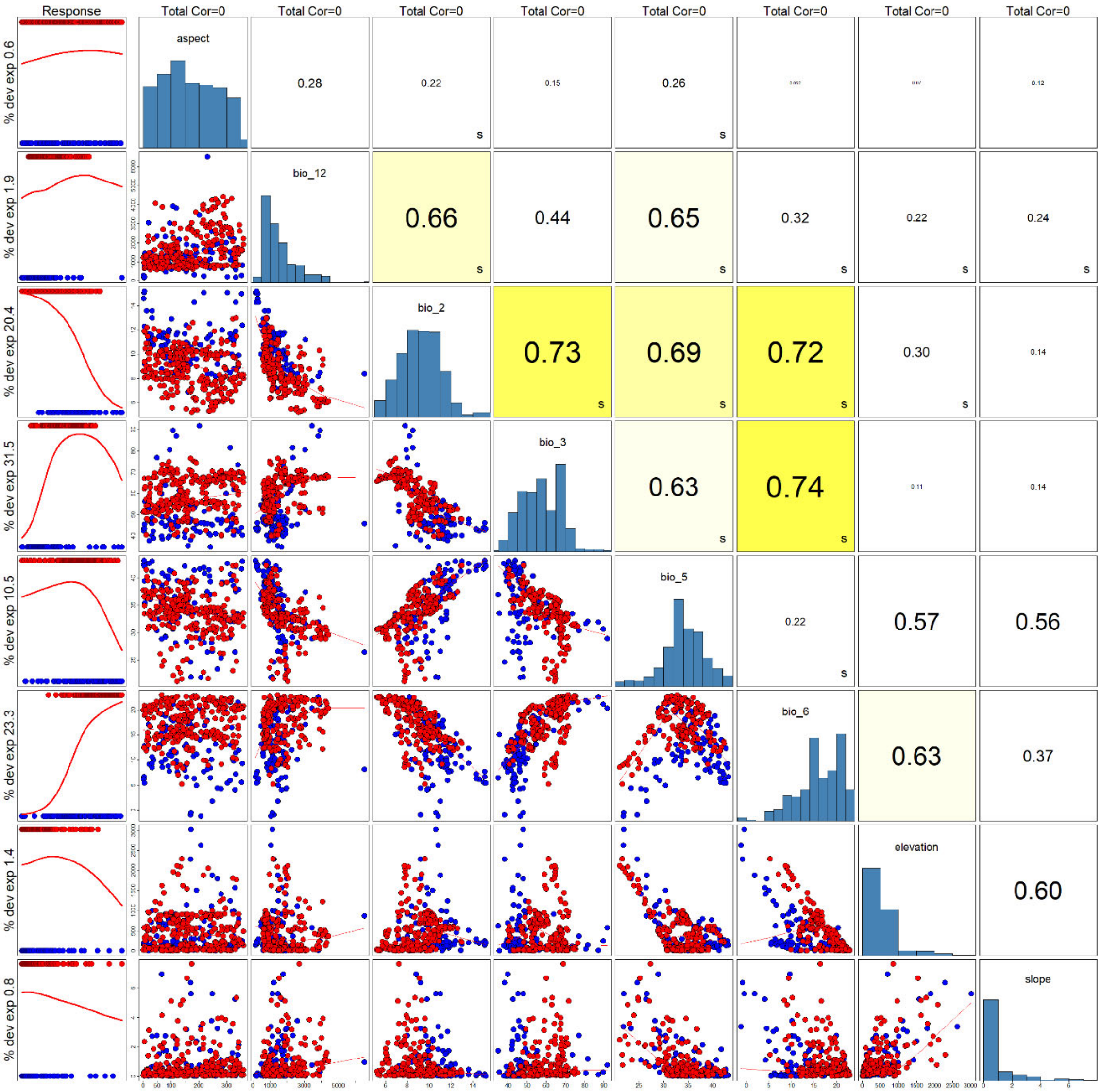

Figure S2. Correlation matrix of covariates selected for the final model of *F. palmarum* (r < 0.8). The Pearson correlation coefficient is primarily shown. If the Spearman or Kendall correlation exceeds the Pearson value, an "s" or "k," respectively, is displayed in the bottom-right corner of the variable box.

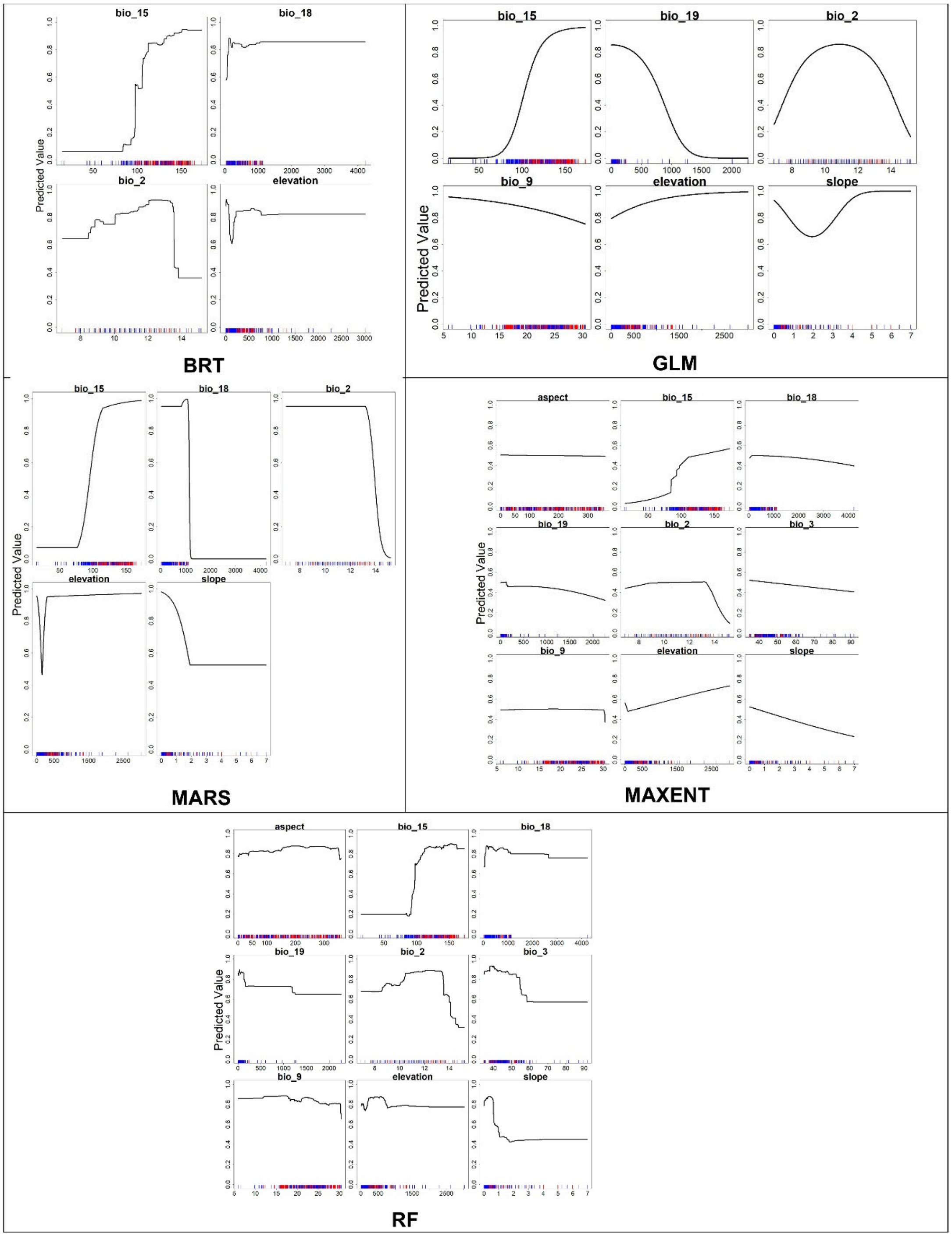

Figure S3. Response curves of each algorithm used in the ensemble model for *F. pennantii*. Each curve illustrates the relationship between predicted habitat suitability and key environmental predictors, as derived from individual algorithms contributing to the ensemble.

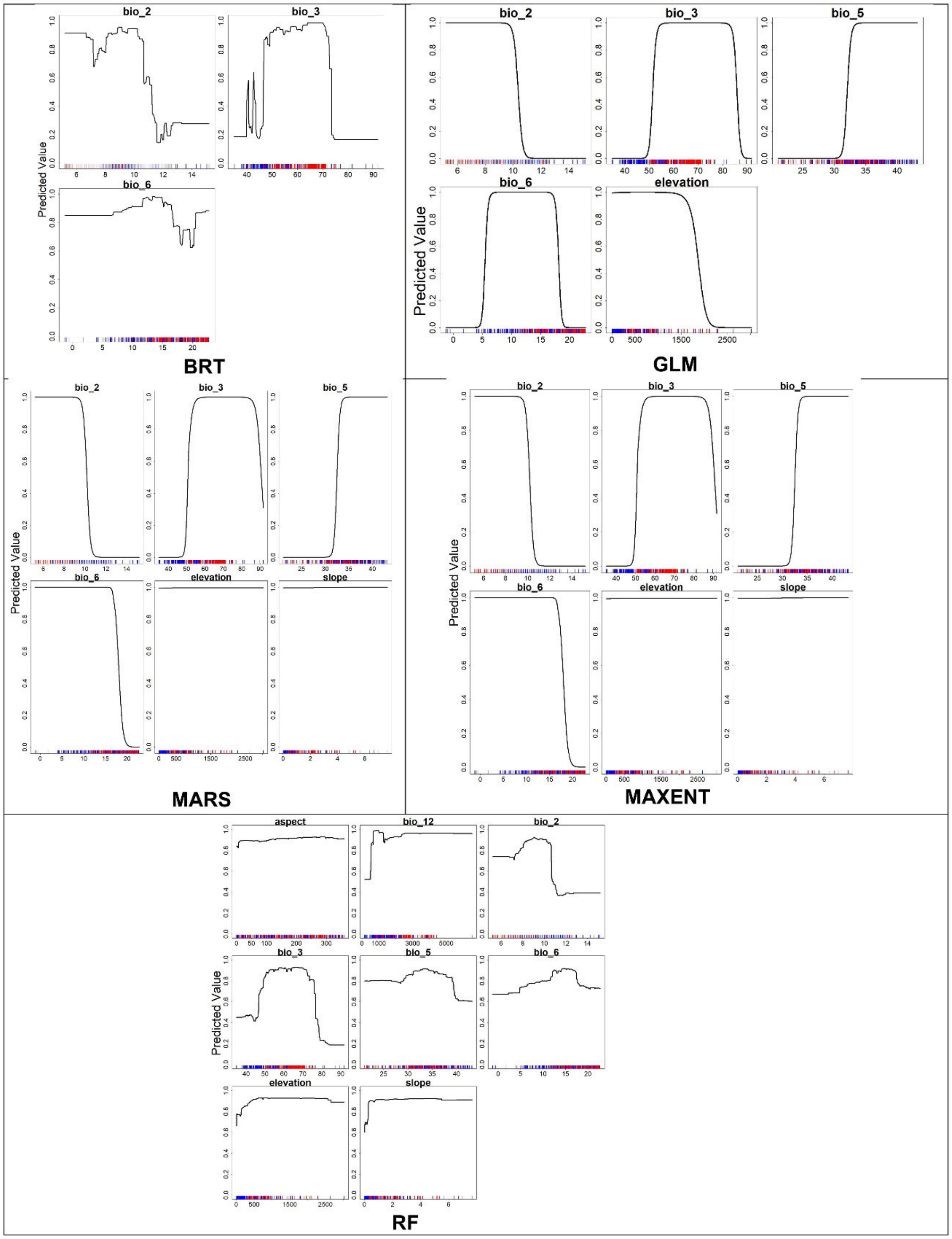

Figure S4. Response curves of each algorithm used in the ensemble model for *F. palmarum*. Each curve illustrates the relationship between predicted habitat suitability and key environmental predictors, as derived from individual algorithms contributing to the ensemble.

Table S1. Suitable habitat area (km²) of *F. pennantii* and *F. palmarum* under present and future climatic scenarios.

Scenario	*F. pennantii*	*F. palmarum*
Present	215748	39578
SSP245 (2041–2060)	313943	65790
SSP245 (2061–2080)	292311	58595
SSP585 (2041–2060)	313344	63311
SSP585 (2061–2080)	260100	79457

Table S2. Indian state-wise area of suitable habitats overlapping with agricultural land for *F. pennantii* under present and future climatic scenarios.

State	Present	SSP245 (2041–2060)	SSP245 (2061–2080)	SSP585 (2041–2060)	SSP585 (2061–2080)
Andaman and Nicobar	0	0	0	0	0
Andhra Pradesh	0	36	126	14	18
Arunachal Pradesh	0	0	0	0	0
Assam	0	0	18	59	0
Bihar	10949	19967	19139	19098	15435
Chandigarh	14	27	27	27	0
Chhattisgarh	11628	28499	28922	28211	27828
Dadra and Nagar Haveli	68	113	99	113	95
Daman and Diu	0	86	72	81	59
Delhi	365	351	365	365	356
Goa	0	176	54	203	9
Gujarat	22766	32477	23274	24921	18459
Haryana	6615	7479	6395	7772	7691
Himachal Pradesh	261	1949	2408	2363	2655
Jammu and Kashmir	68	266	671	446	1422
Jharkhand	6174	15215	12150	14256	13406
Karnataka	657	3641	5130	6300	4023
Kerala	0	0	0	0	0
Lakshadweep	0	0	0	0	0
Madhya Pradesh	48762	37593	32873	37751	16655
Maharashtra	30573	36932	42858	42755	39830
Manipur	0	0	9	0	0
Meghalaya	0	0	0	0	0
Mizoram	0	0	0	0	0
Nagaland	0	0	0	0	0
Orissa	4415	15048	13905	13626	12555
Puducherry	0	0	0	0	0
Punjab	10188	11916	11894	12047	11934
Rajasthan	31973	36122	30852	35609	29385
Sikkim	0	0	0	0	0
Tamil Nadu	0	0	0	0	0
Telangana	8834	10652	13415	11790	10994
Tripura	0	0	0	0	0
Uttar Pradesh	17604	40226	37931	42417	34556
Uttaranchal	1382	3164	2835	3164	3218
West Bengal	2304	11790	6782	9756	9293

Table S3. Indian state-wise area of suitable habitats overlapping with agricultural land for *Funambulus palmarum* under present and future climatic scenarios.

State	Present	SSP245 (2041–2060)	SSP245 (2061–2080)	SSP585 (2041–2060)	SSP585 (2061–2080)
Andaman and Nicobar	0	0	0	0	491
Andhra Pradesh	1472	9603	2970	3690	8294
Arunachal Pradesh	0	0	0	0	0
Assam	5	0	153	90	212
Bihar	0	0	0	0	0
Chandigarh	0	0	0	0	0
Chhattisgarh	0	0	0	0	0
Dadra and Nagar Haveli	0	0	0	0	5
Daman and Diu	0	0	0	0	0
Delhi	0	0	0	0	0
Goa	711	342	711	711	711
Gujarat	18	0	18	18	59
Haryana	0	0	0	0	0
Himachal Pradesh	0	0	0	0	0
Jammu and Kashmir	0	0	0	0	0
Jharkhand	0	0	0	0	0
Karnataka	13262	20079	16281	21663	25524
Kerala	7416	6966	7502	7515	7520
Lakshadweep	0	0	0	0	0
Madhya Pradesh	0	0	0	0	68
Maharashtra	3542	4311	6179	7529	10566
Manipur	0	0	0	0	0
Meghalaya	0	0	0	0	0
Mizoram	0	0	0	0	18
Nagaland	0	0	0	0	0
Orissa	0	0	0	0	0
Puducherry	59	59	86	81	86
Punjab	0	0	0	0	0
Rajasthan	0	0	0	0	0
Sikkim	0	0	0	0	0
Tamil Nadu	12591	23810	24062	21465	25398
Telangana	0	221	0	18	77
Tripura	0	0	0	0	0
Uttar Pradesh	0	0	0	0	0
Uttaranchal	0	0	0	0	0
West Bengal	0	0	113	36	18
